# Blueberry-Enriched Diet Protects Rat Heart from Ischemic Damage

**DOI:** 10.1371/journal.pone.0005954

**Published:** 2009-06-18

**Authors:** Ismayil Ahmet, Edward Spangler, Barbara Shukitt-Hale, Magdalena Juhaszova, Steven J. Sollott, James A. Joseph, Donald K. Ingram, Mark Talan

**Affiliations:** 1 Laboratory of Cardiovascular Science, National Institute on Aging, National Institutes of Health, Baltimore, Maryland, United States of America; 2 Laboratory of Experimental Gerontology, Intramural Research Program, National Institute on Aging, National Institutes of Health, Baltimore, Maryland, United States of America; 3 USDA-ARS, Human Nutrition Research Center on Aging, Tufts University, Boston, Massachusetts, United States of America; 4 Nutritional Neuroscience and Aging Laboratory, Pennington Biomedical Research Center, Louisiana State University System, Baton Rouge, Louisiana, United States of America; Instituto de Química, Universidade de São Paulo, Brazil

## Abstract

**Objectives:**

to assess the cardioprotective properties of a blueberry enriched diet (BD).

**Background:**

Reactive oxygen species (ROS) play a major role in ischemia-related myocardial injury. The attempts to use synthetic antioxidants to block the detrimental effects of ROS have produced mixed or negative results precipitating the interest in natural products. Blueberries are readily available product with the highest antioxidant capacity among fruits and vegetables.

**Methods and Results:**

Following 3-mo of BD or a regular control diet (CD), the threshold for mitochondrial permeability transition (t_MPT_) was measured in isolated cardiomyocytes obtained from young male Fischer-344 rats. Compared to CD, BD resulted in a 24% increase (p<0.001) of ROS indexed t_MPT_. The remaining animals were subjected to a permanent ligation of the left descending coronary artery. 24 hrs later resulting myocardial infarction (MI) in rats on BD was 22% less than in CD rats (p<0.01). Significantly less TUNEL(+) cardiomyocytes (2% vs 9%) and 40% less inflammation cells were observed in the myocardial area at risk of BD compared to CD rats (p<0.01). In the subgroup of rats, after coronary ligation the original diet was either continued or switched to the opposite one, and cardiac remodeling and MI expansion were followed by serial echocardiography for 10 weeks. Measurements suggested that continuation of BD or its withdrawal after MI attenuated or accelerated rates of post MI cardiac remodeling and MI expansion.

**Conclusion:**

A blueberry-enriched diet protected the myocardium from induced ischemic damage and demonstrated the potential to attenuate the development of post MI chronic heart failure.

## Introduction

Reactive oxygen species (ROS) appear to play a major role in ischemia-related myocardial injury [1]–[8]. ROS formation is triggered by depletion of ATP and an overload of Ca^2+^ [2], [4]. In turn, ROS trigger a specific mechanism leading to a change in mitochondrial membrane permeability and eventually to collapse of mitochondrial membrane potential [for review see 9]. It was originally believed that ROS formation occurs primarily, or even exclusively, during reperfusion, when oxygen interacts with the damaged mitochondrial respiratory chain. However, it is now proven in experiments with isolated cardiomyocytes [10] and in the intact hearts [11] that ROS occurs during ischemia from residual O_2_.

The detrimental role of ROS in ischemic and ischemic-reperfusion injury of the myocardium has naturally led to increased interest in antioxidants as therapeutic agents [12]. Unfortunately, thus far, attempts to use synthetic antioxidants to block or attenuate the detrimental effects of ROS have produced mixed and mostly negative results [13]–[15], and increasingly attention has been given to natural products [12].

Blueberries contain anthocyanins, polyphenols and flavonoids and appear to have the highest antioxidant capacity among fruits and vegetables [16]. Blueberry extract and blueberry enriched diets have been shown to reduce age-related behavioral and neuronal deficits in rodents [17]–[20]. Blueberry diets have also inhibited inflammatory cytokines in rat glial cells [21]. Previous research has also indicated that blueberry supplemented rats showed less hippocampal cell loss following experimentally induced stroke [22]. Additionally, it has been reported that blueberry supplemented animals given intra-hippocampal injections of the neurotoxin, kainic acid, showed decreased hippocampal cell loss, reduced cytokine activation, decreased microglial activation and reduced cognitive deficits as compared to non-supplemented kainic acid treated-rats [23], [24]. Therefore, it seems that blueberry supplementation appears to have a tissue-protective effect.

The objective of the current study was to assess the cardioprotective properties of a blueberry enriched diet (BD) in the isolated rat cardiomyocytes and in a rat model of myocardial infarction induced by permanent ligation of a coronary artery. Previously we have used this model to demonstrate the cardioprotective effects of erythropoietin, ˆ_2_-adrenergic receptors agonists, and some dietary manipulations [25]–[27].

## Methods

### Experimental Design

One hundred and fourteen 2-mo old male Fischer-344 rats (Charles River Laboratories Inc., Wilmington, MA) were housed and studied in conformance with the NIH Guide for the Care and Use of Laboratory Animals, Manual 3040–2 (1999), with institutional Animal Care and Use Committee approval. They were randomly divided into two diet groups: regular food (control diet, CD) or blueberry-enriched diet (BD), as described previously [28]. After 3 months on their respective ad libitum diets, 7 CD and 7 BD rats were randomly selected for cardiomyocytes isolation and mitochondrial permeability transition experiments. The remaining animals were subjected to baseline echocardiography followed by a surgical intervention. Following previous protocol [26], 45 rats from each diet group were subjected to a permanent ligation of the left descending coronary artery, and 5 rats from each diet group were sham operated. Among surviving animals, some were sacrificed 24 hrs following surgery, and their hearts were harvested for histological assessment (n = 8 and 9 for BD and CD groups, respectively). Remaining rats were randomly divided into four groups, 9 animals each. Two groups were continued on the same diet they received prior to surgery, and two groups were switched to the opposite diet (blueberry/blueberry, BB; control/control, CC; blueberry/control, BC; and control/blueberry, CB). Additionally, two sham operated groups that had been maintained on blueberry enriched and control diets were continued on the same diet (BBs and CCs). All animals were subjected to repeated echocardiography after 2, 6 and 10 weeks following MI induction. After the last echo test they were euthanized, and their hearts were harvested for histological assessment.

### Diet

The diets were prepared by Harlan Teklad (Madison, WI) using a reformulated NIH-31 diet by adding 20 g/kg lyophilized blueberry (BD) or 20 g/kg dried corn (CD). To prepare the 2% blueberry supplemented diet, blueberries were homogenized in water, centrifuged, lyophilized and added to the NIH-31 rodent chow. The amount of corn in the control diet was adjusted to compensate for the added volume [see 28 for more information on diet preparation and composition]. The two diets were isocaloric within the error margin attributable to routine variations in the nutritional value of the natural ingredients of the NIH-31 diet [29]. The food consumption was determined by subtracting the weight of feed remaining at the end of the week. Based on their observed food consumption during the study, rats maintained on the BD consumed an average of 394 mg/day of lyophilized blueberries, roughly equivalent to 4.4 g/day of fresh blueberries.

### Left Ventricular Myocytes Isolation for Mitochondrial Permeability Transition Experiments

Single ventricular myocytes were isolated via a previously described technique with minor modifications [30]. Briefly, rats were anesthetized with sodium pentobarbital, and hearts were rapidly excised and perfused with 40 ml of nominally Ca^2+^-free bicarbonate buffer gassed with 95% O_2_ - 5% CO_2_ at 37°C. The composition of buffer was the following (in mmol/L): NaCl 116.4, KCl 5.4, MgSO_4_ 1.2, NaH_2_PO_4_ 1.2, glucose 5.6, and NaHCO_3_ 26.2, pH 7.4. Hearts were continuously perfused with bicarbonate buffer containing 0.1% collagenase type B, 0.04 mg/ml protease XVI, and 0.1% BSA type V for 4 min and 50 µmol/L Ca^2+^ was added. After 10 min perfusion, the left ventricle was minced and incubated in bicarbonate buffer containing 100 µmol/L Ca^2+^ for 10 min at 37°C. Myocytes were then resuspended in HEPES buffer with gradually increasing Ca^2+^ concentration up to 1 mmol/L and kept at room temperature until use. The composition of the HEPES buffer was the following (in mmol/L): NaCl 137, KCl 4.9, MgSO_4_ 1.2, NaH_2_PO_4_ 1.2, glucose 15, HEPES 20, and CaCl_2_ 1.0 (adjusted pH to 7.4). Cardiac myocytes viability was typically 70 to 80%.

### Confocal Microscopy and Determination of MPT-ROS Threshold

Experiments were conducted as described previously [31], employing a method to quantify the ROS-susceptibility for the induction of MPT in individual mitochondria within cardiac myocytes [32]. Briefly, isolated cardiac myocytes were exposed *in vitro* to conditions that mimic oxidative stress by repetitive laser scanning of a row of mitochondria in a myocyte loaded with the membrane-potential-sensitive dye, tetramethylrhodamine methyl ester (TMRM). This procedure results in incremental, additive exposure of only the laser-exposed area to the photodynamic production of ROS and consequent MPT induction. The occurrence of MPT induction in a particular mitochondrion is clearly identified by the instantaneous (<1 s) drop in its TMRM fluirescence to background level signifying the immediate and complete dissipation of mitochondrial membrane potential (ΔΨ) causing the loss of its sequestered dye. Myocytes were loaded with 125 nM TMRM for at least 1 h at room temperature and imaged with an LSM-510 inverted confocal microscope (Carl Zeiss Inc., Jena, Germany). Line scan images at 2 Hz were recorded from mitochondria arrayed along individual myofibrils with excitation at 568 nm and collecting emission at >560 nm, using a Zeiss Plan-Apochromat 63×/1.4 N.A. oil immersion objective, and the confocal pinhole was set to obtain spatial resolutions of 0.4 µm in the horizontal plane and 1 µm in the axial dimension. Images were processed by MetaMorph software (Universal Imaging, Downingtown, PA). The ROS threshold for MPT induction (t_MPT_) was measured as the average time necessary to induce MPT in a row consisting of ∼25 mitochondria. Experiments were conducted at 23°C. T_MPT_ was measured in cardiomyocytes isolated from hearts of rats exposed to CD or BD. The cardioprotective action of insulin, which normally results in an enhancement of the MPT-ROS threshold by ∼35–40% [31], was used as a positive control in the present experiments.

### Coronary Artery Ligation

Rats were anesthetized with isoflorene (2% in Oxygen). The surgical procedure was performed as previously described [25]–[27]. Briefly, in aseptic conditions a left thoracotomy was performed in the fifth or sixth intercostal space. The rats were placed and maintained on intermittent positive-pressure ventilation with 95% 02 and 5% CO2. A pericardiotomy was performed and the left descending coronary artery was occluded by snaring and tying a band of myocardium 2–3 mm to the left of the aorta and ligating it with 5-0 silk sutures. Successful ligation was evident by blanching of affected area of myocardium. After chest was surgically closed the residual air was evacuated through a needle puncture. Animal recovered from anesthesia in 10–15 minutes.

### Echocardiography

Echocardiography (Sonos 5500, a 12 MHz transducer) was conducted under light anesthesia by sodium pentobarbital (30 mg/kg, i.p) as previously described [25]–[27]. Briefly, parasternal long axis views were obtained and recorded to ensure that the mitral and aortic valves as well as the apex were visualized. Short axis views were recorded at the mid-papillary muscle level. Endocardial area tracings using the leading edge method were performed in 2D mode (short and long axis views) from digital images captured on cineloop to calculate end-diastolic and end-systolic left ventricular (LV) areas. End-diastolic volume (EDV) and end-systolic volume (ESV) were calculated by a modified Simpson's method. Ejection fraction (EF) was then derived as EF =  (ED−ESV)/ED×100. MI size at the mid-papillary muscle level was estimated from 2D short axis LV images at end-diastole, and expressed as a percent of the LV endocardial circumference. Infarct area was identified as a sharply demarcated section of the LV free wall, which failed to thicken during systole. The length of the akinetic part of the LV endocardial circumference was measured from freeze-frame images at end-diastole. All measurements were made by a single observer blind to the identity of the tracings. All measurements were averaged over three to five consecutive cardiac cycles. The reproducibility of measurements was assessed in two sets of baseline measurements in ten randomly selected rats, and the repeated measure variability did not exceed 5%.

### Histological Acquisition

At 24 hours after MI surgery, a subgroup of rats was euthanized, and their hearts were excised. Using a 16G catheter, 3 ml of 5% Evans blue (Sigma) was rapidly injected into the aorta to distinguish the perfused area (blue staining) from the under-perfused area (no-staining). The atria and great vessels were dissected away from the heart; the heart was cut transversely into four slices from the base to apex. A section from mid-papillary muscle level was immediately stored in liquid nitrogen for later histological analysis. The other three samples were incubated at 37°C with 4% triphenyltetrazolium chloride (TTC, Sigma) for 30 min to distinguish the infarct area (unstained) from the area at risk (AAR; brick red stained) in the under-perfused area. All images were analyzed using NIH Image software. MI size was expressed as a percent of the under-perfused area. Myocardial sections (5 µm thick) were obtained from the frozen sample and stained by Hematoxylene & Eosin (H&E Sigma) and TUNEL (Apop Tag, Chemicon International, Temecula, CA). Inflammatory cells (neutrophils and macrophages) were counted and averaged from the five different fields of the AAR in H&E stained sections in each heart. Apoptosis was assessed from TUNEL stained sections of the AAR from 10 random fields of the AAR. Care was taken to include only clearly identified myocyte nuclei.

Ten weeks after surgery, the remaining rats were euthanized and the hearts were isolated and weighed. The heart weight to BW ratio (HW/BW) was calculated. Myocardial sections (5 µm thick) were obtained from the mid-papillary muscle level. MI size was measured from Masson's trichrome staining sections as the average of infarct area of LV epicardial and endocardial length divided by LV circumferences and expressed as percent of LV. Cardiomyocyte density was averaged from 10 random fields. Cardiomyocyte diameters were measured as a shortest axes through a nucleus.

### Statistical Analysis

All data are expressed as the mean ± SEM. Echo-derived indices were compared pairwise via two-way ANOVA for repeated measurements. Histological and mitochondrial permeability transition data were assessed by one-way ANOVAs. Group differences at specific time-points were tested by Bonferroni = s post-hoc test. (Prism v3.0, GraphPad Software Inc., San Diego, CA). Statistical significance was accepted as P<0.05.

## Results

### Effects of BD on body weight and heart prior to MI

Prior to MI induction, the food consumption and body weight gain did not differ between animals on BD or Control (regular) diet (CD). Starting average body weight was 115 ±3 g in CD rats and 112 ±4 g in BD rats, and after three months reached 351 ±5 and 348 ±5 g in CD and BD, respectively. Echo derived morphometric and functional indices of the heart measured after three months of BD or CD prior to MI induction also did not differ between rats maintained on the two diets (Table 1).

**Table 1:** Echocardiographic indices in rats after 3 months of control or blueberry-enriched diets.

	Blueberry Diet	Control Diet	P
Body Weight (g)	347 ±3	353 ±4	ns
Interventricular septum (mm)	1.06 ±0.02	1.02 ±0.02	ns
Posterior wall thickness (mm)	1.6 ±0.02	1.58 ±0.03	ns
Aortic Diameter (mm)	3.22 ±0.03	3.25 ±0.03	ns
Left Atrial Diameter (mm)	3.64 ±0.09	3.69 ±0.11	ns
LAD/AoD	1.13 ±0.03	1.14 ±0.03	ns
Heart Rate (beat/min)	354 ±5	358 ±5	ns
Fractional Shortening (%)	53.3 ±0.6	52.7 ±0.7	ns
Left Ventricular Mass (g)	0.97 ±0.01	0.96 ±0.01	ns
End Systolic Volume (µL)	105 ±3	106 ±3	ns
End Diastolic Volume (µL)	268 ±5	270 ±5	ns
Stroke Volume (µL)	162 ±3	164 ±4	ns
Ejection Fraction (%)	60.6 ±0.7	60.7 ±0.8	ns
E (cm/sec)	73.9 ±0.9	75.4 ±1.2	ns
A (cm/sec)	52.1 ±1.4	53.0 ±1.2	ns
E/A	144 ±0.05	140 ±0.03	ns
Cardiac Index (µL/g/min)	166 ± 4	163 ± 5	ns

### Assessment of MPT-ROS Threshold

**Figure 1:**
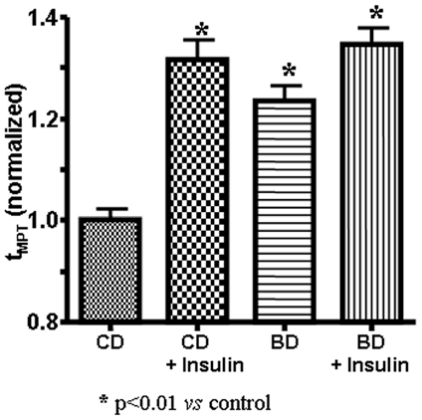
Cellular mechanism of cardioprotection. Blueberry-enriched diet reduces the MPT susceptibility to ROS (t_MPT_) in cardiac myocytes. The t_MPT_ was measured in cells isolated from the hearts of rats, fed for 3 months a control (CD) or blueberry-enriched (BD) diet, before or after exposure to insulin 30 nM (as the positive control) for 20 min. * - p<0.01 *vs* Control.

Figure 1 presents the cardioprotective effects of BD in isolated cardiac myocytes as indexed by the ROS threshold for MPT induction (t_MPT_). Compared to animals on CD, the t_MPT_ was increased in BD rats by 24% (p<0.001). This increase was comparable to an increase induced by insulin, used as a positive control (32%, p>0.05). Addition of insulin to cardiomyocytes from BD rats did not produce any significant increase over and above the increase provided by BD (35%, p>0.05).

### Mortality

Three months of BD had no significant effect on mortality related to ligation of a coronary artery. Perioperative mortality (during the surgery and first 24 hrs after the surgery) was 36% and 40% for the CD and BD groups, respectively (p>0.05). During 10 weeks of observation following MI induction, there was no further mortality, although one CD rat was lost during the echocardiography due to complications of anesthesia.

### 24 hrs following coronary ligation

**Figure 2:**
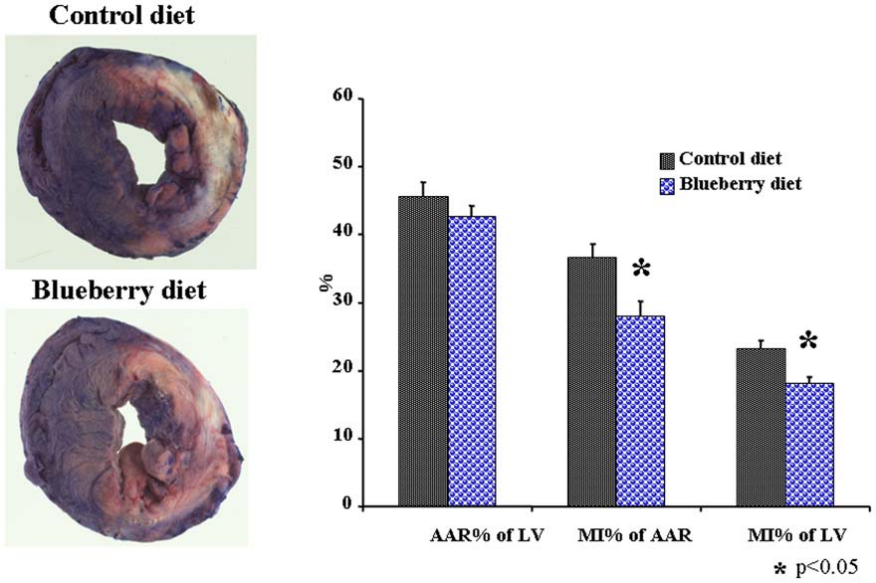
MI 24 hrs following permanent coronary ligation. On the left are samples of the hearts of BD and CD rats subjected to TTC staining: white - necrotic area; red - area at risk (AAR); blue - viable, well-perfused myocardium. On the right is the graph comparing the AAR expressed as a percent of LV and MI size expressed as a percent of LV or AAR in BD and CD rats. *- p<0.01 *vs.* CD.

Figure 2 illustrates the damage to the myocardium 24 hrs after induction of MI in BD and CD animals. Areas at risk (AAR) did not differ between hearts from CD and BD rats. However, the average MI size, calculated either as fraction of AAR or as a fraction of LV, was 22% smaller in BD than in CD rats (p<0.05).

**Figure 3:**
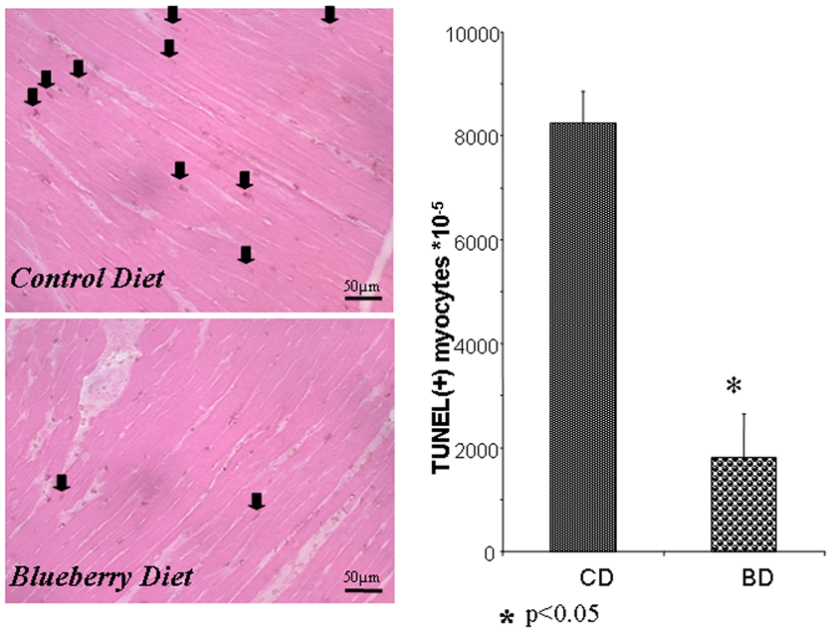
Apoptosis in the myocardial AAR 24-hrs following permanent coronary ligation. TUNEL staining. Number of TUNEL positive cardiomyocyte nuclei are normalized for 10^5^ cardiomyocytes. *- p<0.01 *vs* CD.

TUNEL(+) cardiomyocytes in the AAR are presented in Figure 3. Among CD rats, 9% of cardiomyocytes in the AAR were stained positively for apoptosis (8248 per 10^5^ cardiomyocytes, as shown at Fig. 3), while among BD rats the number of TUNEL(+) cardiomyocytes was reduced to 2% of the total number of cardiomyocytes (p<0.05).

**Figure 4:**
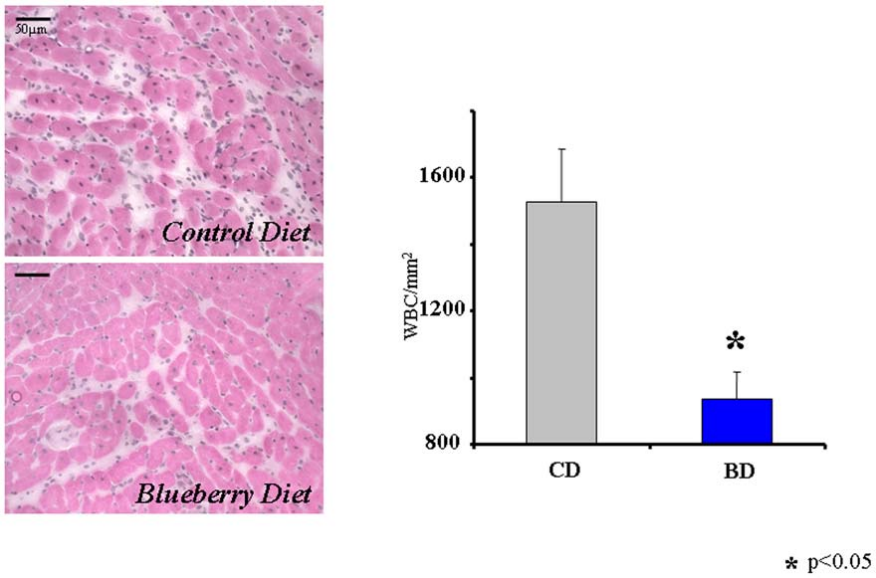
Inflammation in the myocardial AAR 24-hrs following permanent coronary ligation. Hematoxylene & Eosin staining. *- p<0.01 *vs* CD.

The density of inflammatory cells in the AAR is illustrated in Figure 4. In the AAR of BD rats, there were 40% less inflammatory cells (neutrophiles and macrophages) than in the AAR of CD rats (p<0.05).

**To summarize**, 24-hrs following coronary ligation, the BD rats had smaller MI and significantly reduced necro-apoptosis and inflammation in the myocardium surrounding MI than CD rats.

### LV remodeling and function during 10 weeks following MI

The CD and BD rats that survived surgery and were not selected for the acute, 24-hr study were randomly divided into four groups (n = 9 at each group): CD rats that continued on the control diet (CC), BD rats that continued on blueberry enriched diet (BB), BD rats that were switched after surgery from blueberry-enriched to the control diet (BC), and CD rats that were switched from the control to the blueberry-enriched diet (CB). Two groups of sham operated animals were maintained on the same diet as before surgery (BBs and CCs).

**Figure 5:**
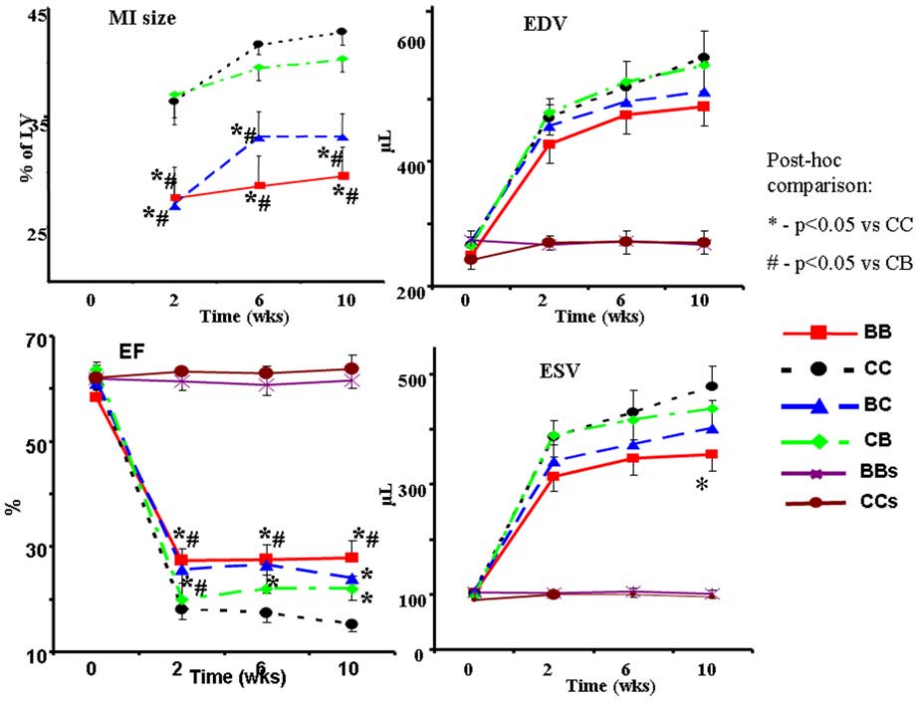
LV remodeling and MI expansion. Echo derived EDV, ESV, EF, and MI size during 10 weeks of post MI observation. Data on sham operated groups (BBs and CCs) is shown for refernce and is not included in statistical comparison. *- p<0.05 *vs.* CC; # - p<0.05 *vs* CB.

Figure 5 illustrates the results of Echo measured progression of LV volumes and MI expansion and functional decline in these four groups from baseline (pre-MI, 0 wks) to 10 weeks following MI induction. The MI size in the original BD groups (BB and BC) was significantly smaller than in CD groups (CC and CB) at each time-point. When BB and CC groups were compared with each other, the ANOVA revealed significant group and time effects, as well as a significant time x group interaction, indicating that in CC group the MI size expanded at a faster rate than in the BB group. However, when BB rats were compared with CB and CC with BC groups, no statistically significant time x group interactions were found. Comparison of Aswitched@ groups (CC vs CB and BB vs BC) showed a trend to slow or to accelerate the MI expansion depending on addition or removal of blueberry, respectively; however the ANOVA derived group x time interaction approached significance (p<0.07) only for the comparison between BB and BC groups, i.e., removal of blueberry from the diet after MI induction accelerated the MI expansion.

The rate of EDV expansion was similar among all groups. However, the statistical evaluation of ESV expansion showed a significant time x group interaction between BB and CC groups, indicating that in CC group the ESV expanded at an accelerated rate compared to BB. However, the time x group interaction was not significant when the BB group was compared with CB and CC was compared with BC groups. Comparison of switched groups showed the tendency of removal or addition of blueberry to the diet to affect the rate of ESV expansion, but the time x group interaction did not reach statistical significance.

Two weeks after MI induction, the EF was significantly higher in BD groups (BB and BC) than in CD groups (CC and CB). In subsequent tests (at 6 and 10 weeks), the EF remained higher in BB rats compared to both CD groups. ANOVA revealed a significant time x group interaction between BB and CC groups, an indication of the difference in the rate of EF decline; however, the interaction was not statistically significant when CC was compared with the BC condition and BB was compared with the CB condition. The interactions between Aswitched@ groups (CC vs CB and BB vs BC) were also not statistically significant. Nevertheless, the differences between BC and CB groups that were statistically significant at the second week disappeared in the subsequent tests due to elevation of EF in CB and its reduction in BC.

**Figure 6:**
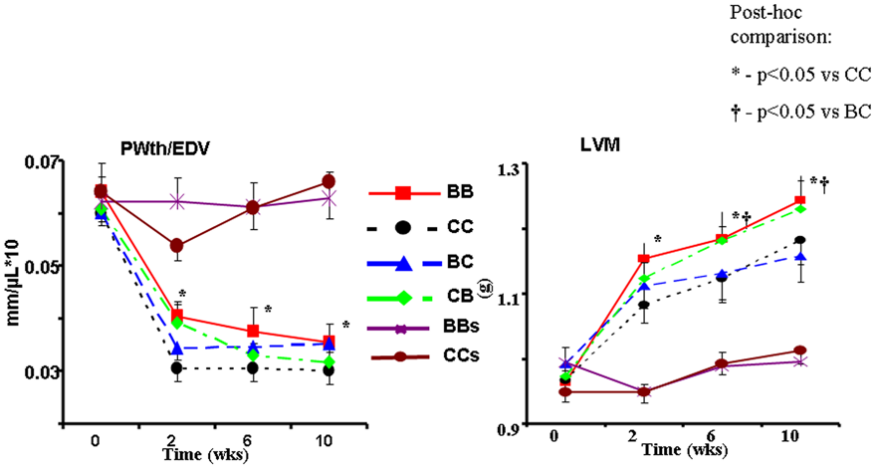
LV hypertrophy. Echo derived measurements of posterior wall thickness normalized for EDV (Pwth/EDV) and LV mass during 10 weeks of post MI observation. Data on sham operated groups (BBs and CCs) is shown for refernce and is not included in statistical comparison. * - p<0.05 *vs* CC; † - p<0.05 *vs* BC.

Figure 6 represents the progression of echo-derived indices of posterior wall thickness (PWth/EDV) and LV mass. All MI animals had significantly thinner PWth normalized for EDV than sham operated animals, however the thinning of the posterior wall was attenuated in BB group compared to CC rats at all time-points. In animals fed blueberry after MI induction (BB and CB), the PWth progressively reduced (p<0.05 for group x time interaction), while in CC and BC groups the PWth did not change or even increased. LV mass was higher in animals fed blueberry-enriched diet following MI (BB and CB) than in the CC and BC conditions.

**To summarize**, comparison of experimental groups, those for which the diet was continued after MI induction with those, for which the diet was switched to the opposite condition, suggests that blueberry-enriched diet might attenuate the post MI cardiac remodeling and MI expansion even when started after MI induction.

**Figure 7:**
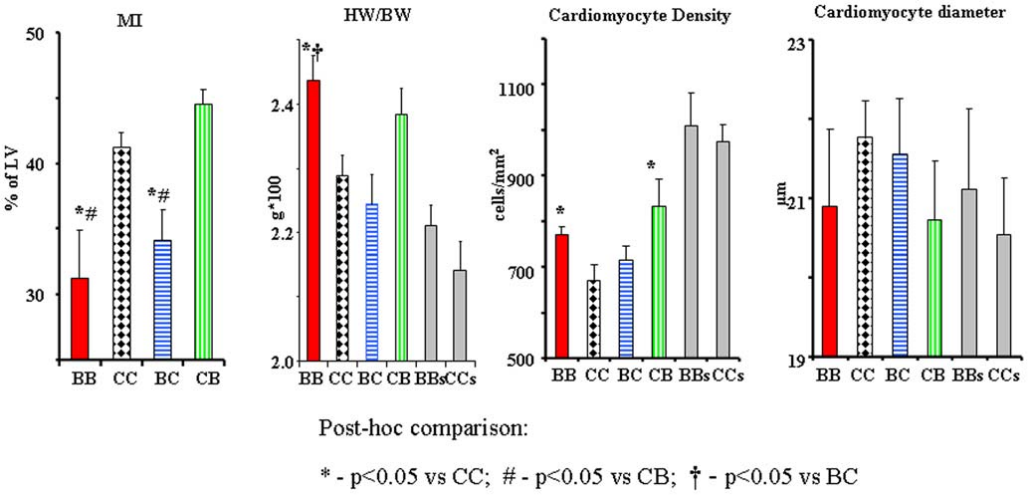
Histological assessment 10 weeks following coronary ligation. From left to right MI size, heart weight normalized for body weight (HW/BW), cardiomyocyte density, and average cardiomyocyte diameter. Data on sham operated groups (BBs and CCs) is shown for refernce and is not included in statistical comparison. * - p<0.05 *vs* CC; # - p<0.05 *vs* CB; † - p<0.05 *vs* BC.

Figure 7 represents the results of histological assessment of the hearts harvested after 10 weeks of post MI observation. Histologically measured MI size was highly correlated (r^2^ = 0.77) with MI size obtained from the last (10 weeks) Echo test. Ten weeks following coronary ligation, the average MI size in both BD groups was significantly smaller than in CD groups (24% difference, p<0.05). The HW/BW ratio was significantly higher in BB group than in both CC and BC. Cardiomyocyte density was reduced in all post-MI animals, but was significantly better preserved among groups fed the blueberry-enriched diet following MI (BB and CB) than in control diet groups (CC and BC). The measurements of single cardiomyocyte diameters did not reveal any significant differences among groups.

## Discussion

The epidemic explosion in the incidence of chronic heart failure (CHF) in Western society resulted in great human suffering and has produced tremendous burden on health care and social services. Since the majority of the CHF develops consequently to MI, the most effective CHF prevention is the prevention of MI from occurrence or minimization of myocardial damage after MI occurrence. The former is targeted through changing life style and diet, leading to reduction of cholesterol and other risk factors, strategies whose arsenal was recently expanded by using pharmacological tools such as statins [33]–[35]. The latter is achieved by rapid and vigorous revascularization and early pharmacological therapy. However, a large fraction of heart attacks is not associated with increased level of cholesterol and its etiology, and thus means of prevention, remain unclear. Therefore, dietary or behavioral manipulations, which could increase the myocardial resistance to ischemic damage, are extremely valuable. Thus far, only exercise has been shown experimentally to increase the resistance of the myocardium to ischemic damage [36]–[38]. We have recently reported, in experiments on rats, that dietary regimen using intermittent, every-other-day fasting significantly increased the myocardial tolerance to ischemic damage [27]. In the present study we have shown for the first time that a regular dietary supplement of blueberry extract attenuates coronary ligation-induced myocardial damage.

Three months of blueberry-enriched diet did not affect food intake or body weight of rats. It also did not have any effect on morphometric or functional echocardiographic indices of the heart. However, experiments with single cardiomyocytes isolated from the LV of BD and CD rats showed significantly increased threshold of ROS-susceptibility for the induction of mitochondrial permeability transition with the blueberry diet. In other words, at the mitochondrial level, the tolerance of cardiomyocytes to oxidative stress was increased with this dietary supplement.

The results of our *in vivo* experiments showed that in the myocardium of rats maintained on a blueberry-enriched diet, 24 hrs after coronary ligation the number of cardiomyocytes in the area at risk stained positively for apoptosis was 4.5 fold reduced compared to that in rats on the control diet; the number of inflammatory cells was reduced almost in half; and MI size resulting from a coronary ligation was 22% smaller. A subset of rats, in which LV remodeling was followed after induction of MI via serial echocardiography, also showed a reduction in original myocardial damage (two weeks after surgery) in BD rats which was naturally translated into significant attenuation of post MI LV remodeling: 10 weeks after coronary ligation rats maintained on blueberry-enriched diet had 14% smaller EDV, 26% smaller ESV, and 82% higher EF than rats fed a control diet. Moreover, 10 weeks after coronary ligation the resulting MI in rats maintained on blueberry-enriched diet prior to and post MI was 31% smaller than in rats maintained on control diet. The positive effect of a blueberry enriched diet post-MI was also confirmed by the assessment of cardiomyocyte density in the myocardium. The reduction of cardiomyocyte density observed 10 weeks after induction of MI in rats maintained on control diet (CC) and reflecting usual loss of cell associated with chronic heart failure [39] was significantly attenuated among BB and CB groups, but was not found in BC group.

This study was designed as a proof of concept and was not intended to analyze the signaling pathways responsible for reduction of necro-apoptosis and inflammation in the myocardium after MI in animals on blueberry-enriched diet. The one thing that could be stated with certainty on the basis of our finding is that this signaling was associated with increased mitochondrial permeability transition threshold. Most probably, based on our previous extensive research, the effect was mediated through a number of possible kinases (31), however the ROS scavenging mechanism also cannot be excluded (32). Some inferences can also be made on the basis of previous studies. Fruits and vegetable rich in polyphenolics are known to delay or reverse the deleterious effects of aging on neurocommunication and behavior [see 40 for review]. Blueberry extract was shown to significantly inhibit the lipopolysacharide-induced inflammatory response in brain microglia. This effect was due to down-regulation of iNOS mRNA and suppression of iNOS proteins, i.e., blueberry extract may inhibit one of the primary steps in the inflammatory stress pathway [41]. In the same model (BV-2 mouse microglial cells), blueberry extract treatment also inhibited COX-2 mRNA and protein expression [41], which is known to be associated with proinflammatory stimuli [42], [43], and the proinflammatory cytokines IL-1β and TNF-α [41]. Extrapolating from these findings, it is reasonable to assume that in the present study similar oxidative/inflammatory stress signals may be operational. Much more difficult is to explain the reduced necro-apoptosis in our model. Blueberry-enriched diet is known to have some anti-cancer properties [44], [45] and one of the anti-cancer mechanisms is the activation of apoptosis [46].

An additional promising direction in understanding the tissue-protective properties of blueberry might be in exploring the possible effects of BB treatment on glucose metabolism and, specifically, its insulin-like properties [47], [48]. Tissue-protective properties of insulin are expanded well beyond its control of hyperglycemia. It suppresses the production of TNF-α, IL-6, and other pro-inflammatory cytokines and enhances the synthesis of anti-inflammatory cytokines, IL-4 and IL-10 [for review see 49]. Specifically, cardioprotective effects of insulin were demonstrated clinically [50] and in experimental models [51]. Multiple mechanisms of cardioprotection were proposed: coronary dilatation, anti-inflammatory and anti-apoptotic; however, the most fundamental to cardioprotection remained insulin = s ability to stabilize the mitochondrial permeability transition (52). Thus, it is conceivable that increased myocardial tolerance to hypoxia after prolonged feeding with a blueberry-enriched diet, as well as elevation of mitochondrial permeability transition threshold shown in our study, are due to up-regulation of insulin sensitivity.

Therefore, the Apreventive@ part of our experiment clearly demonstrated that blueberry-enriched diet increased tolerance to ischemic damage of myocardium in a rat model of a permanent coronary ligation. The Atreatment@ part of the study, however, while less obvious, is also very promising. The switching of the diet immediately after coronary ligation to its opposite, i.e., blueberry-enriched to control and control to blueberry-enriched, revealed a tendency to affect the post MI progression of LV remodeling by accelerating or attenuating it respectively. This outcome indicates the possibility of adding blueberry supplementation to the therapeutic arsenal which should be evaluated further in experimental models of CHF.

**In summary**, in experiments examining rats maintained on a blueberry enriched diet, we found increased myocardial tolerance to ischemic damage. At a cellular level, the blueberry diet increased cardiomyocyte survival by elevating the mitochondrial permeability transition ROS threshold. In *in vivo* experiments this diet reduced the size of myocardial infarction induced by a permanent coronary ligation by attenuating necro-apoptosis and inflammation in the area at risk. The beneficial effects of the blueberry diet were extended after coronary ligation by continuing the attenuation of the post-MI LV remodeling and MI expansion. The only non-pharmacological intervention capable to produce similar cardioprotective effect so far was the intermittent fasting (27). To the best of our knowledge, this is the first demonstration of the effectiveness of a readily available natural product in acceptable quantity to significantly limit myocardial damage resulted from induced ischemia.
